# Budapest nephrology school – 30 years of history – from modest start to an international success: systematic summary of the 27th BNS held between 28^th^ August and 2^nd^ of September 2023

**DOI:** 10.1080/0886022X.2023.2282709

**Published:** 2024-04-29

**Authors:** Orsolya Cseprekál, Laszlo Rosivall

**Affiliations:** a Department of Surgery, Transplantation and Gastroenterology, Semmelweis University, Budapest, Hungary; b Institute of Translational Medicine, International Nephrology Research and Training Center, Semmelweis University, Budapest, Hungary

**Keywords:** Education, nephrology, Budapest, translational medicine

## Abstract

Budapest Nephrology School (BNS) could have celebrated its 30th event if it had not been interrupted by COVID pandemic for a few years. Yet, the organization of 27th BNS in August 2023 resumed its successful and traditional activities at Semmelweis University, in the beautiful central European city of Budapest. In over two decades, BNS has faithfully adapted to the changes and developments of medical science and clinical nephrology, the fact which has kept it unique and attractive for nephrologists from across the globe. With such a long history and representing the top international professors of nephrology, BNS has proved to be a successful one-week, in-person refreshing course which has attracted over 1600 medical doctors from more than 60 countries. It has well served as an academic meeting point suitable for networking and exchange of up-to-date knowledge presented by the best international experts in nephrology. The dedication and focus of these experts on education, research and patient care represent the very concept of translational medicine. The invaluable experience of the past 27 years has set the standards for BNS to contribute to the evolution of translational nephrology in Europe in the next decade.

## Where does it come from?

At the beginning of the twentieth century, Sándor von Korányi, Hungarian doctor coined and explained first the term “renal insufficiency” by applying the freezing point reduction to measure osmotic activity of urine [1, 2]. He analyzed renal function by measuring its concentrating ability and hence explained “renal insufficiency” and hyposthenuria. This was considered as a major milestone in the evolution of nephrology [3]. Hungarian nephrology remained traditionally strong in both clinical and research fields. However, due to unfavorable political and human conditions in the early ‘80s, the development of nephrology in Hungary experienced a slowdown compared to the international trends, yet the general standards of practice remained higher in Hungary with respect to other Eastern and Central European countries. Understanding the problems of the time, László Rosivall as a young dedicated doctor was determined to bring about the very much-needed change in Hungarian nephrology by establishing a kidney foundation and inviting the help of his mentors and friends in the West [4]. He was convinced that a real change was vital and knew the revival of Hungarian nephrology was possible only *via* reinforced, continuous and systematic research and clinical activities. He first embarked on training himself and gained invaluable knowledge and experience while working in different laboratories of University of Bergen (with Knut Auckland), University of Alabama (with G.L. Navar), and University of Heidelberg (with Wilhelm Kriz and Roland Taugner) among others. Soon after his return to Hungary, he founded the **Hungarian Kidney Foundation** (1987) which was then the first health centered nonprofit charity organization in Hungary dedicated to support the development of nephrology in Hungary and other Central and Eastern European countries. By supporting education, research and clinical practice, Hungarian Kidney Foundation set the Hungarian nephrology back in the right track and initiated its development. These supports and grants resulted in many interdepartmental collaborations, studies, scholarships and networking at European and international levels. The Hungarian Kidney Foundation also established the “Nephrology Research and Training Centre” and the first nephrology PhD School in Hungary (1993) at Semmelweis University under the supervision of Dr. Rosivall. This PhD school has successfully trained many young scientists with 71 degrees conferred so far, of which 33 were directly guided by Dr. Rosivall.

On several occasions, Dr. Rosivall held discussions with the renowned Roscoe Robinson “Ike”, who was then the president of ISN and editor-in-chief of Kidney International. Rosivall asked Robinson for his support to establish the Budapest Nephrology School (BNS) that could bring huge benefits for ex-socialist countries in the region [5]. The argument was that BNS would contribute to the revitalization of Hungarian nephrology by training the young talented nephrologists who can later become teachers for BNS. The idea proved to be true and effective which attracted more and more audience year by year. The 27th BNS was a good testimony when a number of Hungarian professors from western universities came to Budapest to lecture and share their knowledge with the audience from many different countries. BNS is both effective in terms of academic content and also in financial terms compared to other CME courses. BNS is affordable and it is located in a place that makes it accessible for any young medical doctor from Europe and overseas. It has been one of the main goals of BNS to train a critical number of young nephrologists who can later play considerable roles in their countries and regions to support the development of nephrology and increase the standards of patient care. As of today, over 1600 physicians have benefited from a well-structured postgraduate level nephrology course at BNS in Budapest. Within a few years, BNS contributions resulted in the establishment of “International Nephrology Research and Training Center” at Semmelweis University. This center requested the cooperation of the best nephrology professionals from all over the world. Thank to its three decades of constant work, the center has trained a remarkable number of young nephrologists from Hungary and other countries. This center has supported some of its young trainees to go to the USA, Canada and Germany for further education at a partner university. Both BNS and the center have focused on educating talented young researchers and clinicians who will, in turn, undertake educating other younger physicians. Hungarian and regional nephrology was again on the radar after a long period of silence and sleep. It was in 2003, when Dr. Rosivall wrote a letter to the then president of IFKF (International Federation of Kindey Foundations) explaining the success of BNS and his ideas for a better European and international nephrology. In part of his letter, he wrote: ***“I would like to make a recommendation for our organization in order to have more recognition and support worldwide for kidney diseases, research and renal education. I think it is time and it is our duty to call for the attention of the nations, governments and statesmen to announce and support a Day or a “Week of KIDNEY”***. The World Kidney Day (WKD) has been organized and celebrated since 2005.

The Budapest Nephrology School (BNS) has taken the long and successful path of development since 1993 [6]. An article was published on the 20th anniversary of BNS [7]. As of today, young physicians from 68 countries have attended BNS, namely from Albania, Algeria, Austria, Bahrain, Belarus, Belgium, Bosnia and Herzegovina, Brazil, Bulgaria, Canada, China, Croatia, Czech Republic, Denmark, Ecuador, Egypt, England, Estonia, France, Germany, Greece, Hungary, India, Indonesia, Iran, Israel, Italy, Japan, Jordan, Jugoslavia, Kasachstan, Kenya, Latvia, Libya, Lithuania, Macedonia, Moldova, Mongolia, Montenegro, Netherlands, New Zealand, Nigeria, Norway, Pakistan, Poland, Portugal, Qatar, Romania, Russia, Serbia, Slovakia, Slovenia, South Africa, Sri Lanka, Sweden, Switzerland, Turkey, USA, Ukraine, United Kingdom, Venezuela.

BNS is also supported by the Hungarian Society of Nephrology, and endorsed by the European Renal Association (ERA) and the International Society of Nephrology (ISN). The faculty of BNS has always been from the best experts who rightly deserve to be named here, some of these great teachers are not among us any more,: T. Andreoli, J. Balla, J. M. Bargman, C. Baylis, P. Bárány, P. D. Bell, W. van Biesen, R. Blantz, B. M. Brenner, M. E. De Broe, R. de Châtel, D. Cohen, E. Cole, A. J. Collins, R. Coppo, W. Couser, A. Covic, P. Csermely, A. Davison, Gy. Deák, G. Devins, J. Dirks, T. B. Drücke, Cs. Dzsinich, K-U. Eckardt, A. Falus, Cs. Farsang, J. Feehally, M. Fischereder, L. G. Fine, J. Floege, A. Fogo, D. Fouque, M. Geiszt, P. Gergely, D. Goldsmith, M. S. Goligorsky, S. Goral, J. G. Grunfeld, S. Halperin, Á. Haris, L. Harper, U. Heemann, A. Heidland, J. H. Helderman, G. Hercz, L. Hunyadi, E. J. Holtzman, A. Horváth, A. Iaina, N. Ismail, B. Iványi, K. Jager, J. Járay, V-M. Kahari, K. Kauser, D. Kerjaschki, M. Ketteler, J. T. Kielstein, I. Kiss, H. Klinkmann, E. Kolossváry, G. Kovács, Cs. P. Kovesdy, R. T. Krediet, K. Kurokawa, S. Van Laecke, N. Lameire, R. Langer, A. Logan, N. Levin, M. Little, F. Locatelli, G. M. London, F. Luft, A. MacLeod, G. Mayer, O. Mehls, D. C. Mendelssohn, A. Meyrier, I. Mucsi, S. Mustata, G. A. Müller, H. Mürer, J. Nagy, D. Naimark, S. Nielsen, M. Novák, R. Oberbauer, K. Olgaard, H. H. Parving, M. Paul, F. Perner, J. Peti-Peterdi, R. Pisoni, K. Polner, J. Rees, E. Ritz, B. Rodríguez-Iturbe, C. Ronco, P. Ronco, L. Rosivall, B. Rutkowski, Rychlik, I, Sadayoshi, B. Sarkadi, D. Sclöndorff, H. Schmidt-Gayk, G. Schulman, K. Skorecki, E.Slatopolsky, S. Sonkodi, G. Spasovski, Spät, Z. Stevanovic, T. Szabó, B. Szamosfalvi, M. Tapolyai, V. Tesar, Tislér, J. Titze, S. W. Tobe, T. Tóth, T. Tulassay, R. Vanholder, S. Vas, P. Venetianer, J. J. Weening, A. Wiecek, I. Wittman, D. De Zeeuw, P. Å. Zillén.

## What about the 27th BNS?

As the first post-COVID event, between August 28 and September 2 of 2023, the 27th BNS was organized by the Hungarian Kidney Foundation at the Semmelweis University (Department of Pathology and Experimental Cancer Research). The Young Committee of the Hungarian Society of Nephrology (MANET) also actively participated in the management. Following its traditions, the 27th BNS was endorsed by the European Renal Association (ERA) and the International Society of Nephrology (ISN).

A friendly motto goes among young nephrologists saying *"anyone who wants to be a leader in this profession must perform first here"*. Interestingly, as the first post-COVID event, many European participants (including Slovenia, Albania, Romania, Lithuania, etc.) and non-Europeans as far as Indonesia and Australia attended the course this year. Hungarian Kidney Foundation provided some scholarships to support the participation of young Hungarian specialists and residents. The course has been accredited by EACCME (European Accreditation Council for Continuing Medical Education) and OFTEX (Hungarian Continuing Medical Education Portal), to provide added values and certificates which can be taken home.

The scientific program of the 27th BNS covered the most exciting and innovative areas of nephrology, from molecules to the bedside. Among reviewed subjects, the newly discovered regulatory mechanisms of the salt-water household and its interaction with metabolism, the role of blood pressure and the central nervous system, the latest tools for monitoring kidney function at the molecular level, glomerular diseases, transplantation, dialysis, and rare diseases were thoroughly discussed. This 6-days intensive scientific course was further enriched by colorful social programs such as wine-tasting dinner, wine competition, Danube cruise, organ concert, exploring the main building of Semmelweis University, a tour in the Hungarian Parliament, and thai-chi, complemented with the Hungarian hospitality.

This year our lecturers were: Ottó Árkossy, Germany; Jan U. Becker, Germany; Marc E. De Broe, Belgium; Szilveszter Dolgos, Hungary; Simin Goral, USA; Georgina Gyarmati, USA; Ágnes Haris, Hungary; J. Harold Helderman, USA; Csaba P Kovesdy, USA; Friedrich C. Luft, Germany; Miklos Z Molnar, USA; György Nagy, Hungary; Pontus B. Persson, Germany; János Peti Peterdi, USA; Zoltán Prohászka, Hungary; György Reusz, Hungary; László Rosivall, Hungary; András Tislér, Hungary; Jens Marc Titze, Singapur; Kálmán Tory, Hungary; and Sotirios G. Zarogiannis, Greece.

Nothing is more rewarding than to witness the happiness and content of our young colleagues who attend the course. This is a sign that BNS should and will continue to educate the young generation of nephrologists in the future. We quote what a colleague from Indonesia wrote to the President of BNS: “*I wanted to take a moment to express my heartfelt gratitude for the incredible experience I had at Budapest Nephrology School. It was an honor and a privilege to attend this prestigious event and I am immensely grateful for the warmth and welcome I received from You and the entire faculty. The expertise of the faculties were truly exceptional. The opportunity to learn from and interact with such well-known international experts in the field of nephrology was a dream come true. I would also like to extend my appreciation to the organizing committee for their meticulous planning and dedication to ensuring the success of the event did not go unnoticed. I was particularly pleased to find that my spouse and child could attend the social events without any problems. This experience has left a lasting impact and I’m excited to apply the knowledge gained to benefit nephrology in my home country. Thank you*”.

## Present of BNS – where are we now?

COVID pandemic did leave unprecedented effects on the world which greatly affected education, training and interpersonal communications. For a while, it seemed that online education will totally overtake other forms of education, making it unnecessary to hold personal meetings or travel. Meantime, the speed by which new information is generated and passed over has tremendously increased. It is all dazzling and confusing. Do we need a personal meeting in such an era and environment?! The 27th BNS gave the answer. We cannot and do not want to go back in time, nor can we slow down the changes, but in this fast developing world, we want to preserve the most important elements of personal relationships. BNS could successfully continue its journey in 2023 and is now planning for the next decade to both incorporate the technological changes brought by AI and embrace the human side of interaction and education in an elite environment of an international nephrology school.

## Future – where are we going? (Quo vadis BNS?)

Budapest Nephrology School will adapt to the fast technological and academic changes in the world and in the next decade continue playing its role in training and education of young nephrologists. BNS will employ a holistic approach covering subjects from molecules to bedside care. BNS will continue applying translational approach in education and it will continue bringing the best experts of the field and the young doctors under the same roof for a productive, intensive and joyful course. The 28^th^ BNS will be held between *August 26-31. 2024* in Budapest, Hungary.
